# Biomechanical force profiles and force decrease of direct printed versus thermoformed aligners with different trimlines: an in vitro analysis

**DOI:** 10.1186/s40510-026-00627-0

**Published:** 2026-06-03

**Authors:** Bernhard Wiechens, Philipp Meyer-Marcotty, Emilia von Waldthausen, Lisa-Marie Mai, Jonas Q. Schmid, Phillipp Brockmeyer, Wolfram Hahn, Anja Quast

**Affiliations:** 1 Department of Orthodontics, Universitätsmedizin Göttingen, Göttingen, Germany; 2 Department of Orthodontics, University of Münster, Münster, Germany; 3 Department of Oral and Maxillofacial Surgery, Universitätsmedizin Göttingen, Göttingen, Germany

**Keywords:** Direct printed aligners, Thermoformed aligners, Force decrease, Vertical force, Gingival margin design, Orthodontics

## Abstract

**Background:**

This in-vitro study aimed to evaluate and compare the biomechanical force profiles of thermoformed and direct-printed aligners with different gingival trimlines during facial and palatal translation of a maxillary central incisor.

**Methods:**

A 3D-printed model with a mounted central incisor was used to simulate 0.25 mm of bodily movement. Forces were recorded in three axes (Fx, Fy, Fz) using a multi-axis force sensor. Three aligner types (thermoformed with straight trimline: TFA_S_, and direct-printed with either straight: DPA_S_ or garlanded trimline: DPA_G_) were tested (*n* = 10 each). Vertical, transverse, and sagittal force components and their decrease were analyzed. Forces were recorded over 60 min to characterize initial force delivery and early stress relaxation under standardized in vitro conditions.

**Results:**

Thermoformed aligners exhibited the highest sagittal forces during facial movement (Fx − 0.70 N), while DPA_G_ demonstrated significantly lower forces (Fx − 0.25 N; *p* < .001). During palatal translation, DPA_S_ reached peak forces of 0.57 N, whereas DPA_G_ showed lower, more controlled forces (*p* < .001). Vertical forces (Fz) were significantly higher in TFA_S_ (− 0.10 N), while DPA remained near zero or slightly extrusive (*p* < .001).

**Conclusions:**

In this in vitro model, DPA, particularly DPA_G_, showed lower sagittal force values and smaller vertical force components. In the first 60 min, DPA showed a pronounced early force decrease. Longer observation periods are required to describe force behavior beyond the initial seating phase.

## Introduction

Significant advances in material science have enabled the direct fabrication of clear aligners using CAD-CAM technology. This innovation promises significant economic benefits and potential improvements in biomechanical performance [1, 2]. Until now, conventional thermoformed aligners (TFA) have struggled with notable geometric inaccuracies, dimensional variations, low mechanical strength, and poor wear resistance [3]. These issues are attributed to both the materials used and the multi-step thermoforming process. Some aligner materials also have very narrow processing temperature ranges; exceeding these ranges can lead to material oxidation, compromising aligner transparency and aesthetics [4].

In addition to material properties, aligner thickness has a critical influence on the biomechanics and resulting force delivery. Several studies have indicated that thicker aligners tend to exert higher forces [5, 6]. However, thermoforming often results in inconsistent thickness - thicker in the posterior than in the anterior regions, and thinner facially than palatally [7]. Since aligner thickness determines force profiles and deflection behaviour, this variability significantly limits biomechanical predictability [3]. The resulting lack of precision can impair clinical outcomes and is one reason why the overall accuracy of tooth movement using conventional TFA is only estimated at 46–56% [8].

Moreover, TFA have been shown to generate forces three to eleven times higher than ranges reported for conventional appliances, especially during incisor tipping [6]. Increased forces have been associated with patient discomfort and complications including root resorption or hyalinization [9].

Although orthodontic forces have been discussed extensively, there is no consensus on a single ideal force magnitude for specific tooth movements, and reported ranges differ by model, appliance system, and outcome definitions.

In contrast, directly printed aligners (DPA) represent a novel approach with the potential to overcome many of the limitations inherent to TFA [10, 11]. Using additive manufacturing, DPA are fabricated from photocurable resins such as TC-85, which possess thermo-mechanical properties favourable for orthodontic applications, including shape memory and elastic resilience [12, 13]. These properties allow DPA to deliver forces that are more consistent and directionally controlled. Moreover, DPA can be precisely customized with uniform thickness and trimlines tailored to the biomechanics of specific tooth movements [14].

However, little is known about how specific design elements - especially gingival trimline morphology - affect the magnitude and direction of generated forces. The garlanded margins, in particular, may offer biomechanical advantages through improved edge flexibility and optimized force distribution [15]. Additionally, the initial time-dependent relaxation behaviour (force decrease over 60 min) of directly printed aligners has not been fully explored under standardized laboratory conditions [16, 17].

This study therefore aimed to investigate the force magnitude, direction, and decrease over time (60 min) of thermoformed and directly printed aligners with different trimlines during in-vitro translation of a maxillary central incisor. The goal is to evaluate how material properties and trimlines affect initial biomechanical output and to identify design strategies that may improve the predictability of force vectors in this model. Therefore, the null hypothesis of the present study was: DPA show no differences in their biomechanical properties compared to thermoformed aligners, considering both gingival trimline design and simulated occlusal loading.

## Materials and methods

**Fig. 1:**
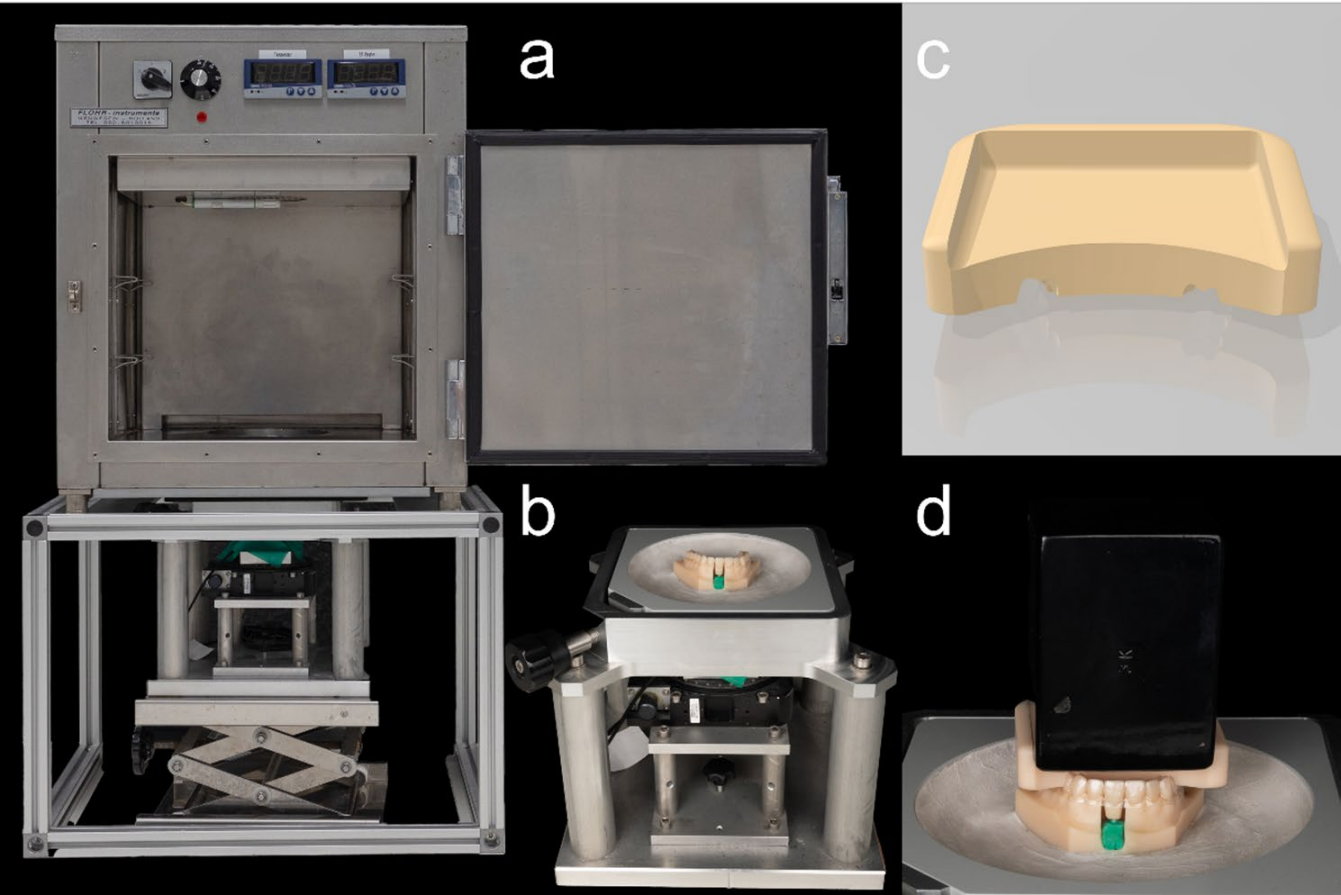
Test setup. Complete test setup with **a** incubator, **b** sensor unit and **c** and **d** loading device

This study employed a validated in-vitro test system [5, 6] using a multi-axis force sensor (Fig. 1b; Nano 17, ATI Industrial Automation, USA) integrated into a rigid optimized maxillary training model (Frasaco GmbH, Germany) with a modular central incisor (tooth 21). The model was captured and virtualized via intraoral scanning (TRIOS 4, 3shape, USA).

**Fig. 2:**
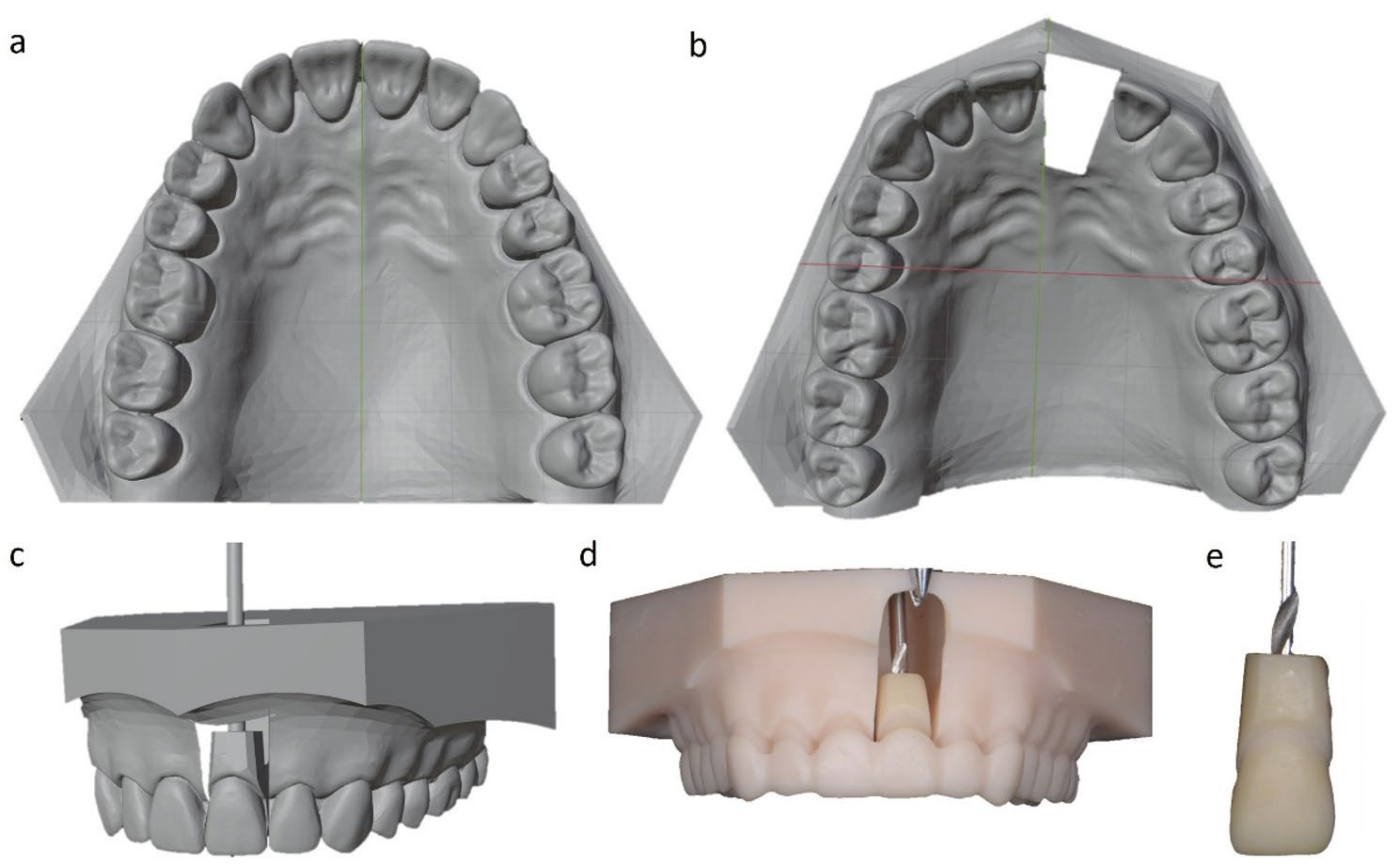
CAD-CAM of investigation unit. **a** unsegmented virtualized model, **b** segmented virtualized model jaw, **c** virtualized model jaw with a separated measuring tooth and planned mounting pin, **d** 3D printed model jaw with inserted mounting pin and mounting aid and **e** measuring tooth with inserted mounting pin

Thereafter software-assisted segmentation and drilling of the sample tooth as well as model preparation for sensor unit integration (Blender v. 4.0.2, Blender Foundation, Netherlands) was performed. The incisor was programmed to undergo 0.25 mm of bodily translation either facially or palatally, simulating a clinically relevant movement scenario. After 3D printing of the investigation base, the measuring tooth and a virtually planned mounting aid (Fig. 2; OnyxCeph, Image Instruments, Germany), the set-up was cemented in a resin bowl (Fig. 1a, b,d; GC Fujirock^®^ EP, GC GERMANY GmbH, Germany).

**Fig. 3:**
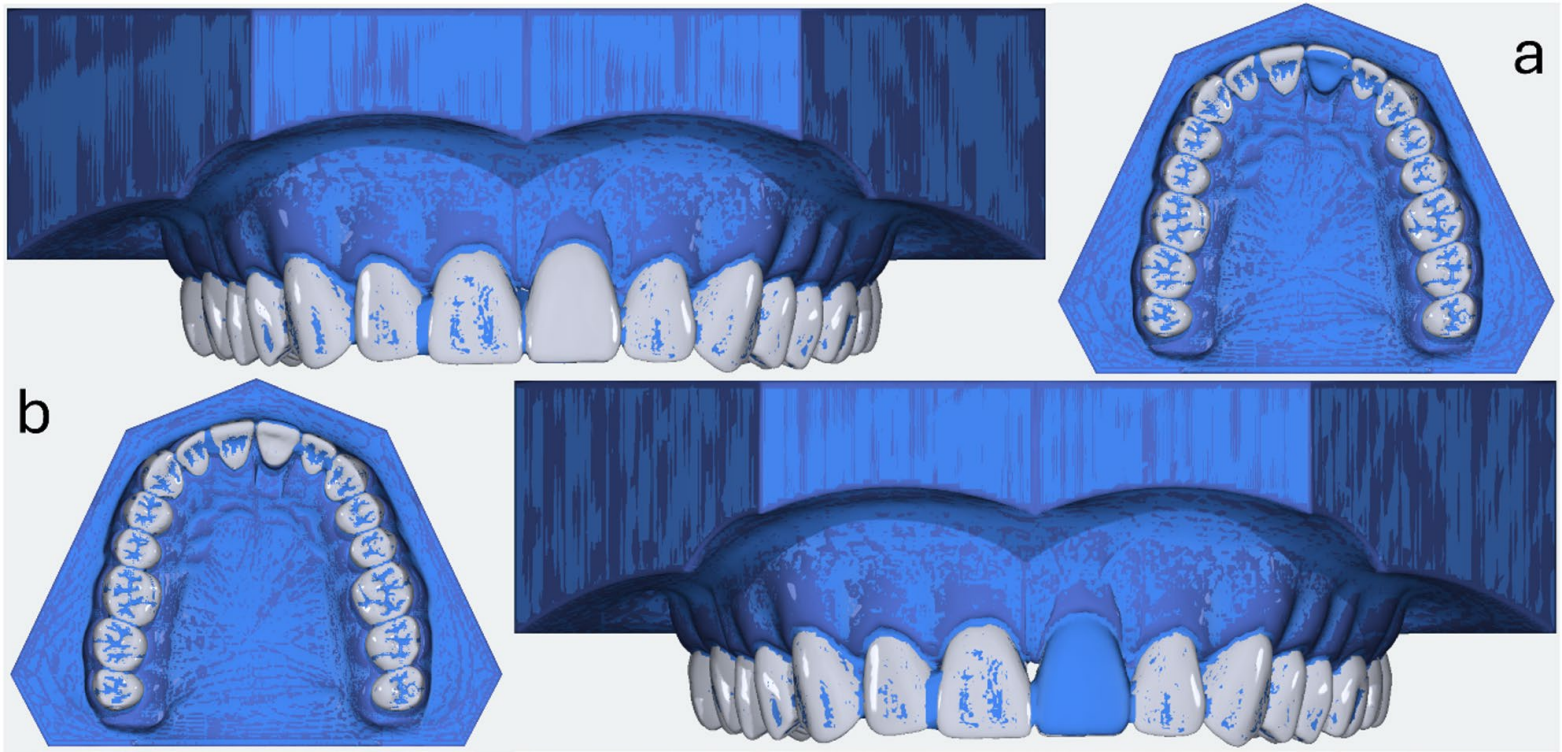
Superimposition of intended range of motion. **a** palatal movement, **b** facial movement

Measurements were conducted in an incubator (Fig. 1a; Flohr Instruments, Netherlands) at 36.5° Celsius under application of artificial saliva (Saseem, G. Pohl-Boskamp GmbH & Co. KG, Germany). The sensor was calibrated following manufacturer specifications with 1% full-scale accuracy. After installation, another scan was performed for virtual reconstruction and planning of the tooth movement (Fig. 3; Aligner 3D, OnyxCeph, Image Instruments, Germany). In accordance with step-size recommendations for a clinical treatment step, a 0.25 mm vestibular and palatal translation was planned (Fig. 3a and b) [18].

**Fig. 4:**
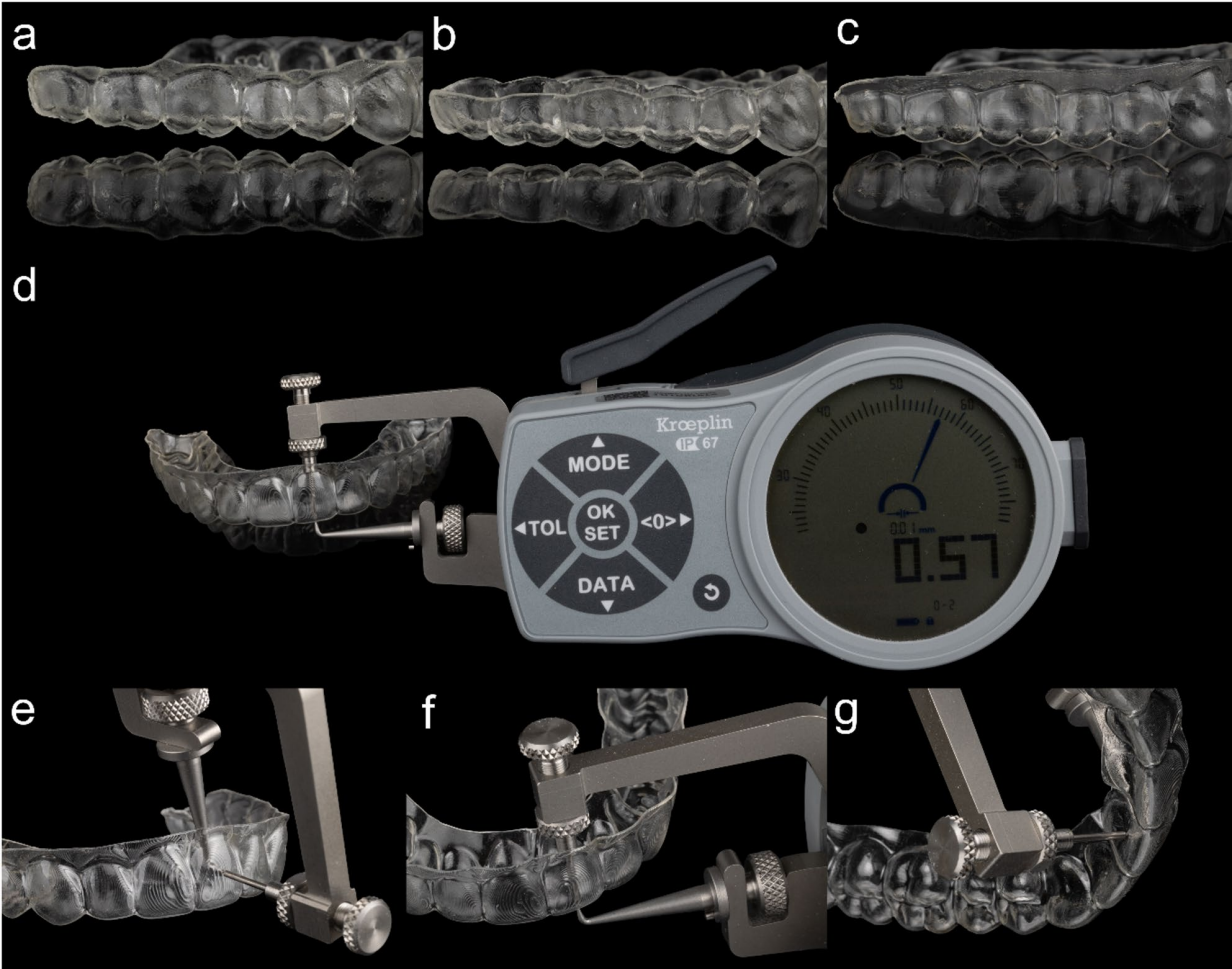
Tested and measured samples. Illustration of aligners examined: **a** DPA_S_, **b** DPA_G_, and **c** TFA_S_. Custom-made measuring instrument (**d**). Demonstration of measurement points: **e** facial, **f** incisal and **g** palatal

Subsequently, a .stl file was generated for printing the modified dental arch to fabricate the TFA. The thermoformed aligners (Duran 0.75 mm, SCHEU-DENTAL GmbH, Germany) ended up exactly at the model boundary. The planning for DPA production was also carried out virtually with a straight (DPA_S_) and a garland-shaped (DPA_G_) aligner border (Bitesplint 3D, OnyxCeph, Image Instruments, Germany). The planned material thickness of the aligners was 0.75 mm. The planned vertical extension of the straight trimmed aligners was + 1.5 mm while the vertical extension of the garland-trimmed aligners was − 1 mm. Corresponding .stl files were generated and sent to an external production department (Fig. 4; Graphy Inc., Republic of Korea). As TC-85DAC is an in-house material produced by the developer (Graphy Inc., Republic of Korea), regulatory compliance with manufacturing standards was assumed.

Ten aligners were produced for each group, i.e. TFA_S_, DPA_S_ and DPA_G_. Due to the material properties of DPA, the force measurements of all examined samples were carried out at the following measuring times in minutes: 0,1,2,3,4,5 and then at 5-minute intervals over an hour. The 60-minute observation period aimed to quantify initial force delivery and early relaxation after seating. Additional measurements were performed under simulated chewing force of 3 kg. For this purpose, a hollow weight tray was designed (Blender v. 4.0.2, Blender Foundation, Netherlands) and a 3 kg weight (Sport-Thieme GmbH, Germany) inserted, which was positioned on the posterior support zones only (without direct contact to the measuring tooth) to standardize seating and simulate occlusal loading during aligner wear; forces were recorded using the same time schedule as in the unloaded condition (Fig. 1c and d). One of the ten aligners from the TFA group was examined according to the measurement protocol described the others after 60 min. The rigid sensor unit recorded the resulting force values in Newtons (N) in the sagittal (x), transverse (y) and vertical (z) planes. Positive signs in the sagittal plane coded for oral-directed, in the transverse plane for distal-directed and in the vertical plane for extrusive-directed force vectors, vice versa. Finally, the thickness of all examined aligners was measured using an electronic external measuring device (K1 × 095 custom-made, Kroeplin Längenmesstechnik, Germany) in the center of facial, incisal and palatal surfaces of the measuring tooth region.

### Statistical analysis

Descriptive data analysis included normality testing using the Shapiro-Wilk tests. Normally distributed variables were reported as means and standard deviations (SD). Non-normally distributed variables were reported as medians and interquartile ranges (IQR). Parametric data were analysed using one-way analysis of variance (ANOVA). Non-parametric data were analysed using Kruskal-Wallis test and Dunn´s multiple comparisons tests. Due to the nature of the statistical analysis, p-value adjustment (corr.p.) for repeated measurements was performed according to Bonferroni. All analyses were performed using GraphPad Prism 10 (v. 10.2.3, GraphPad Software, USA) at a significance level of α = 5%.

## Results

### Sagittal forces (Fx)

During facial translation, sagittal force magnitudes were highest in TFA_S_, followed by DPA_S_ and DPA_G_ (Table 1). Group differences were significant, with TFA_S_ showing the largest intrusive components.

In palatal translation, all groups generated positive sagittal forces, with TFA_S_ again showing the highest magnitudes and both DPA groups expressing lower values (Table 2). Differences between groups were significant in the overall comparison.

### Vertical forces (Fz)

During facial translation, TFA_S_ exhibited higher intrusive vertical forces than both DPA groups, which remained close to zero (Table 1).

In palatal translation, TFA_S_ produced clearly higher vertical force components, whereas DPA_S_ and DPA_G_ showed much smaller vertical effects with opposite directions of force vectors (Table 2).

### Transverse forces (Fy)

Transverse forces were generally small compared with the sagittal components but differed between groups. During facial translation, TFA_S_ and DPA_S_ tended toward mesial forces, while DPA_G_ showed slightly distal-directed components (Table 1).

In palatal translation, the DPA groups differed in direction, with DPA_S_ showing mesial and DPA_G_ distal tendencies, and TFA_S_ remaining slightly distal; overall group differences were significant (Table 2).

**Table 1:** Descriptive and analytic statistics of facial force magnitudes

Resulting forces facial translation		TFA_S_ (*n* = 10)	DPA_S_ (*n* = 10)	DPA_G_ (*n* = 10)	Overall group comparison	Intergroup comparison	TFA_S_ (*n* = 10)	DPA_S_ (*n* = 10)	DPA_G_ (*n* = 10)	Overall group comparison	Intergroup comparison
		Straight	Straight	Garlanded	(ANOVA)	TFA_S_ vs. DPA_S_	TFA_S_ vs. DPA_G_	DPA_S_ vs. DPA_G_	Straight	Straight	Garlanded
	Unit	Mean	SD	Mean	SD	Mean	SD	p	corr.p	corr.p	corr.p
Sagittal (x)	cN	-57.37	44.92	-15.95	7.42	-3.47	8.86	< 0.001	0.005	< 0.001	ns
Transverse (y)	cN	-2.01	8.82	-1.38	6.10	5.19	5.55	0.057	ns	ns	ns
Vertical (z)	cN	-7.28	14.36	-3.64	5.65	-2.60	6.27	0.535	ns	ns	ns

Corresponding results of ANOVA followed by Bonferroni’s multiple comparisons test between groups after 60 min. Means and standard deviations (SD) of centinewtons (cN) were reported; significance level was set at *p* < .05 and adjusted by Bonferroni correction (corr.p). ns = not significant

**Table 2:** Descriptive and analytic statistics of palatal force magnitudes

Resulting forces palatal translation	TFA_S_ (*n* = 10)	DPA_S_ (*n* = 10)	DPA_G_ (*n* = 10)	Overall group comparison	Intergroup comparison	TFA_S_ (*n* = 10)	DPA_S_ (*n* = 10)	DPA_G_ (*n* = 10)	Overall group comparison	Intergroup comparison	TFA_S_ (*n* = 10)
		Straight	Straight	Garlanded	(ANOVA)	TFA_S_ vs. DPA_S_	TFA_S_ vs. DPA_G_	DPA_S_ vs. DPA_G_	Straight	Straight	Garlanded
	Unit	Mean	SD	Mean	SD	Mean	SD	p	corr.p	corr.p	corr.p
Sagittal (x)	cN	60.79	1.69	30.22	10.18	25.77	6.41	< 0.001	< 0.001	< 0.001	.502^ns^
Transverse (y)	cN	4.38	3.46	-2.96	4.99	6.27	3.48	< 0.001	0.001	.912^ns^	< 0.001
Vertical (z)	cN	39.80	5.84	1.73	2.49	-5.34	3.19	< 0.001	< 0.001	< 0.001	0.002

Corresponding results of ANOVA followed by Bonferroni’s multiple comparisons test between groups after 60 min. Means and standard deviations (SD) of centinewtons (cN) were reported; significance level was set at *p* < .05 and adjusted by Bonferroni correction (corr.p). ns = not significant

### Force decrease behavior

**Fig. 5:**
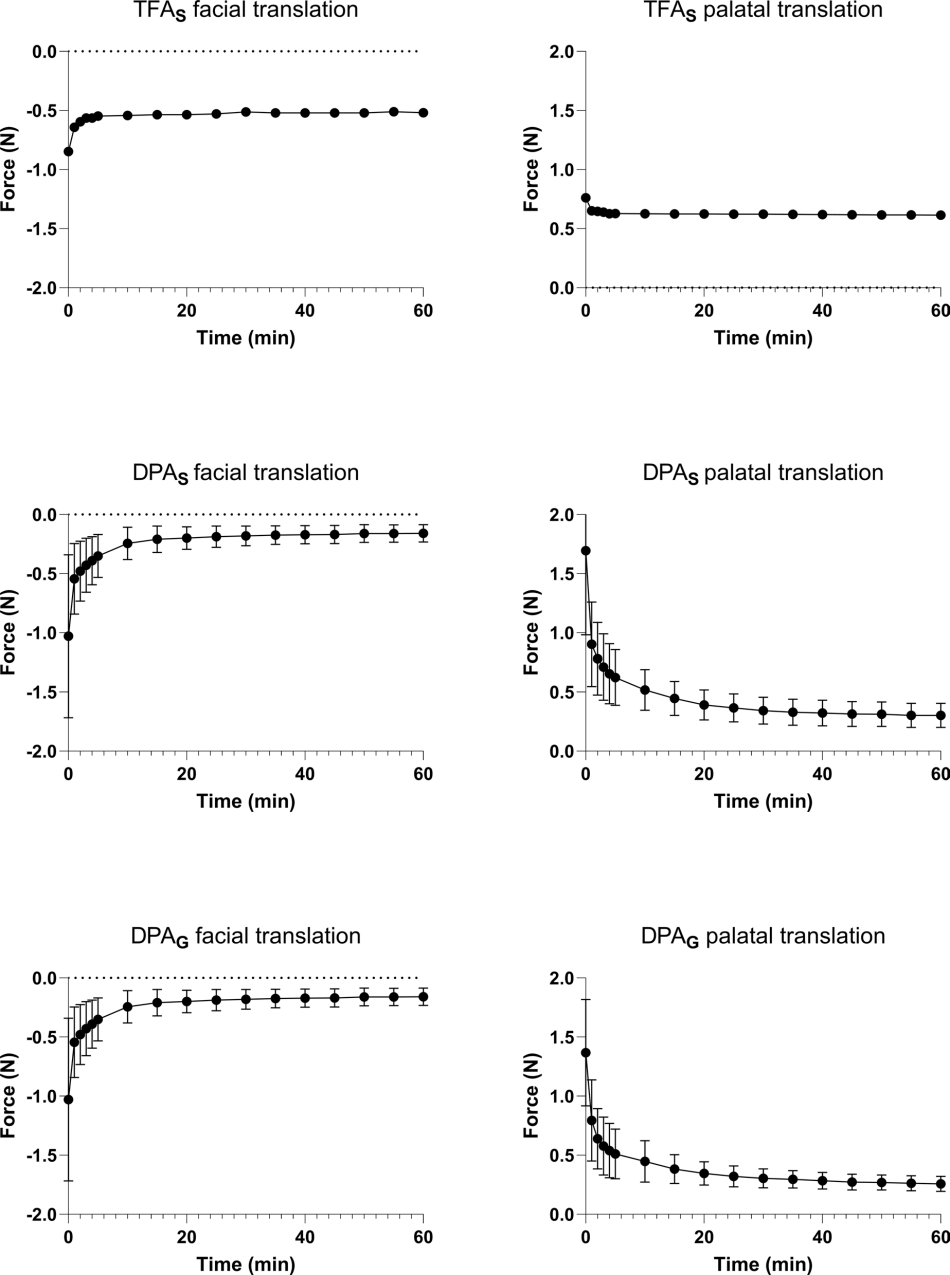
Longitudinal force development. Diagram shows means (dots) of force development with standard deviations (whiskers) over 60 min in Newtons (N)

Across all groups, the highest force magnitudes occurred immediately after activation and decreased over time. In facial translation, TFA_S_ showed a steep initial peak followed by a gradual decline, whereas DPA_G_ exhibited a smoother, near-linear reduction and DPA_S_ remained comparatively low throughout the 60-minute period (Fig. 5).

In palatal translation, force decrease was most pronounced in the DPA groups, particularly in DPA_G_, which showed a rapid early decline. TFA_S_ retained higher residual forces at the end of the 60-minute observation period (Fig. 5).

### Influence of loading and occlusal simulation

**Fig. 6:**
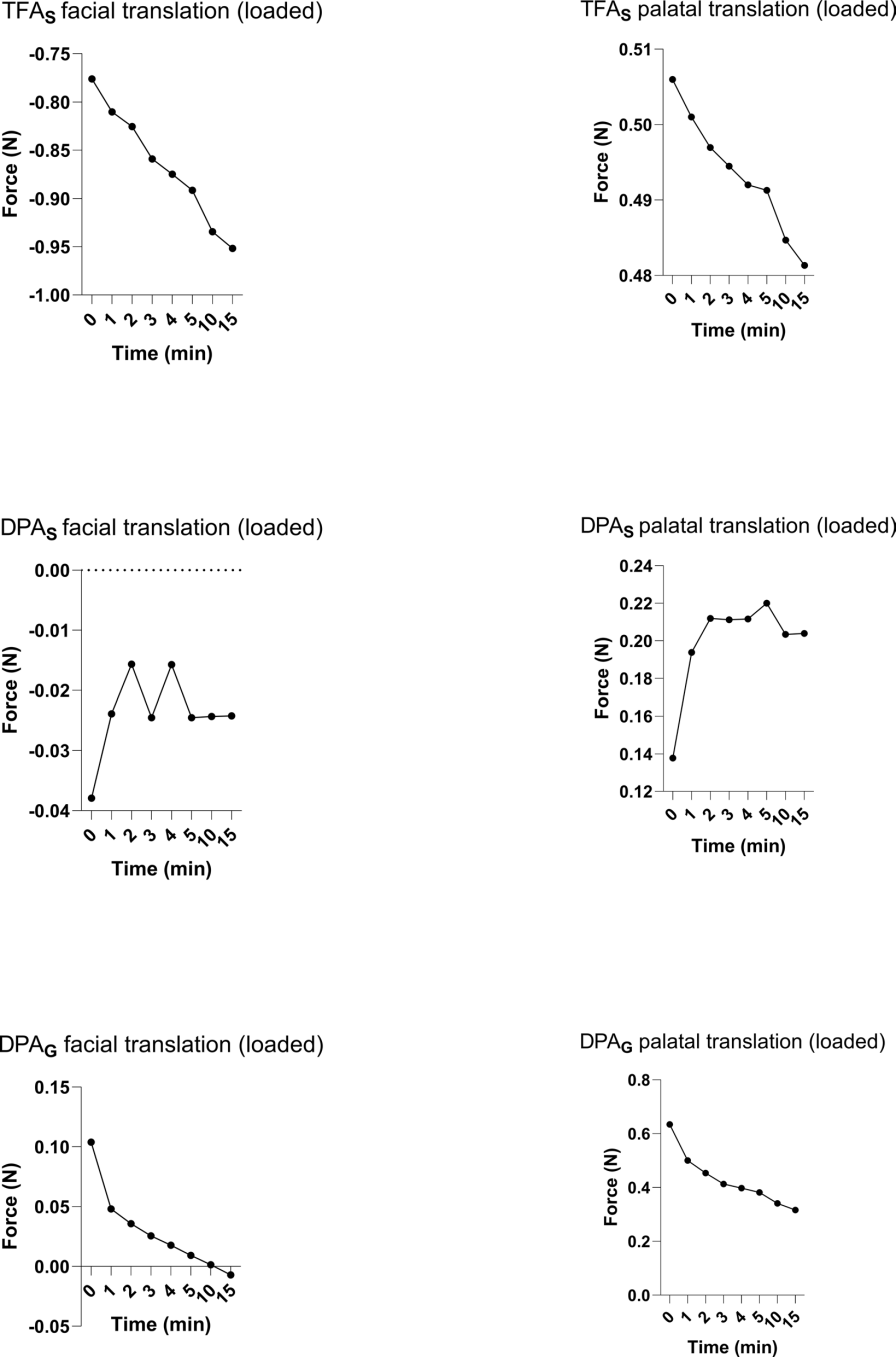
Longitudinal force development under loading. Diagram shows forces under 3 kg of loading over 15 min in Newtons (N)

Under simulated occlusal loading during facial translation (Fig. 6), TFA_S_ generated clearly higher sagittal and vertical force components than DPA_S_ and DPA_G_ (Table 3). The printed aligners showed comparatively small sagittal forces and limited vertical components in this condition.

In loaded palatal translation (Fig. 6), TFA_S_ again produced higher sagittal forces than DPA_S_, while DPA_G_ reached intermediate values (Table 4). Vertical and transverse components differed between groups, indicating that both material and trimline geometry influenced the force system under loading.

**Table 3:** Descriptive and analytic statistics of loaded facial force magnitudes

Resulting forces facial translation loaded		TFA_S_ (*n* = 10)	DPA_S_ (*n* = 10)	DPA_G_ (*n* = 10)	Overall group comparison	Intergroup comparison	TFA_S_ (*n* = 10)	DPA_S_ (*n* = 10)	DPA_G_ (*n* = 10)	Overall group comparison	Intergroup comparison
		Straight	Straight	Garlanded	(ANOVA)	TFA_S_ vs. DPA_S_	TFA_S_ vs. DPA_G_	DPA_S_ vs. DPA_G_	Straight	Straight	Garlanded
	Unit	Mean	SD	Mean	SD	Mean	SD	p	corr.p	corr.p	corr.p
Sagittal (x)	cN	-86.55	6.05	-2.38	0.69	2.93	3.51	< 0.001	< 0.001	< 0.001	0.048
Transverse (y)	cN	4.23	3.32	9.70	4.84	-4.03	2.53	< 0.001	0.022	< 0.001	< 0.001
Vertical (z)	cN	-83.61	11.61	-3.97	2.72	-13.51	9.03	< 0.001	< 0.001	< 0.001	.115^ns^

Corresponding results of ANOVA followed by Bonferroni’s multiple comparisons test between groups after 60 min. Means and standard differences (SD) of centinewtons (cN) were reported; significance level was set at *p* < .05 and adjusted by Bonferroni correction (corr.p). ns = not significant

**Table 4:** Descriptive and analytic statistics of loaded palatal force magnitudes

Resulting forces palatal translation loaded		TFA_S_ (*n* = 10)	DPA_S_ (*n* = 10)	DPA_G_ (*n* = 10)	Overall group comparison	Intergroup comparison	TFA_S_ (*n* = 10)	DPA_S_ (*n* = 10)	DPA_G_ (*n* = 10)	Overall group comparison	Intergroup comparison
		Straight	Straight	Garlanded	(ANOVA)	TFA_S_ vs. DPA_S_	TFA_S_ vs. DPA_G_	DPA_S_ vs. DPA_G_	Straight	Straight	Garlanded
	Unit	Mean	SD	Mean	SD	Mean	SD	p	corr.p	corr.p	corr.p
Sagittal (x)	cN	49.35	0.81	19.92	2.60	42.98	10.13	< 0.001	< 0.001	.142^ns^	< 0.001
Transverse (y)	cN	3.06	0.29	-14.71	16.22	7.12	1.67	< 0.001	0.003	> .999^ns^	< 0.001
Vertical (z)	cN	-3.61	2.99	0.48	2.25	-18.00	5.44	< 0.001	.132^ns^	< 0.001	< 0.001

Corresponding results of ANOVA followed by Bonferroni’s multiple comparisons test between groups after 60 min. Means and standard differences (SD) of centinewtons (cN) were reported; significance level was set at *p* < .05 and adjusted by Bonferroni correction (corr.p). ns = not significant

### Aligner thickness

TFA_S_ showed lower thickness values at all three measurement points (facial, incisal, palatal) compared with both DPA groups, which exhibited greater and more variable thicknesses (Tables 5 and 6). Despite this, the printed groups generated lower sagittal and vertical force magnitudes than TFA_S_, indicating that trimline design and material behavior contributed substantially to the observed force profiles.

**Table 5:** Descriptive and analytic statistics of facial translation aligners dimensions

Diameters facial translation aligners		TFA_S_ (*n* = 10)	DPA_S_ (*n* = 10)	DPA_G_ (*n* = 10)	Overall group comparison	Intergroup comparison	TFA_S_ (*n* = 10)	DPA_S_ (*n* = 10)	DPA_G_ (*n* = 10)	Overall group comparison	Intergroup comparison
		Straight	Straight	Garlanded	(Kruskal Wallis)	TFA_S_ vs. DPA_S_	TFA_S_ vs. DPA_G_	DPA_S_ vs. DPA_G_	straight	Straight	Garlanded
	Unit	Median	IQR	Median	IQR	Median	IQR	p	corr.p	corr.p	corr.p
Facial surface	mm	0.42	0	0.75	0.02	0.67	0.07	< 0.001	< 0.001	0.025	0.048
Incisal surface	mm	0.60	0.02	0.77	0.05	0.68	0.04	< 0.001	< 0.001	.057^ns^	.051^ns^
Palatal surface	mm	0.65	0.02	0.99	0.18	0.77	0.09	< 0.001	< 0.001	0.032	0.032

Corresponding results of Kruskal-Wallis and Dunn´s multiple comparisons tests. Medians and interquartile ranges (IQR) of millimeters (mm) were reported; significance level was set at *p* < .05 and adjusted by Bonferroni correction (corr.p). ns = not significant

**Table 6:** Descriptive and analytic statistics of palatal translation aligners dimensions

Diameters palatal translation aligners		TFA_S_ (*n* = 10)			DPA_S_ (*n* = 10)		DPA_G_ (*n* = 10)		Overall group comparison	Intergroup comparison		
		Straight			Straight		Garlanded		(Kruskal Wallis)	TFA_S_ vs. DPA_S_	TFA_S_ vs. DPA_G_	DPA_S_ vs. DPA_G_
	Unit	Median	IQR	Median		IQR	Median	IQR	*p*	corr.*p*	corr.*p*	corr.*p*
Facial surface	mm	0.42	0.01		0.75	0.10	0.69	0.09	< 0.001	< 0.001	0.021	.068^ns^
Incisal surface	mm	0.62	0.03		0.74	0.0	0.66	0.10	< 0.001	< 0.001	0.049	.172^ns^
Palatal surface	mm	0.63	0.02		0.95	0.07	0.78	0.05	< 0.001	< 0.001	0.032	0.032

Corresponding results of Kruskal-Wallis and Dunn´s multiple comparisons tests. Medians and interquartile ranges (IQR) of millimeters (mm) were reported; significance level was set at *p* < .05 and adjusted by Bonferroni correction (corr.p). ns = not significant

## Discussion

Within the limitations of this in vitro model DPA showed lower sagittal force magnitudes and reduced vertical force components compared with thermoformed aligners. Early force decrease over the first 60 min was more pronounced in the printed groups. In particular, the DPA_G_ group exhibited lower force magnitudes falling within published experimental ranges, characterized by a steady degressive force decrease, low initial peak forces, and reduced side effects in vertical and transverse dimensions [13, 16].

This study observed that TFA_S_ generated sagittal forces approximately two to three times higher than the DPA groups, a finding that aligns with previous literature on overactivation in thermoformed materials [6, 19]. High initial loads are considered to carry the risk of exceeding the ranges for tooth movement specified in the literature, which may increase the risk of side effects such as root resorption or pain [9]. In contrast, the forces produced by DPA_G_ remained within clinically reported limits (< 0.30 N) [20], even immediately upon insertion, indicating that design optimization can help mitigate excessive loading.

Vertical force vectors (Fz) were almost completely absent in DPA_G_ and DPA_S_. This was noteworthy as intrusive or extrusive forces associated with translational motion are rarely intentional. This supports the hypothesis that trimlines play a crucial role in vector control, a finding confirmed by Elshazly et al. [15], who found that garlanded and extended margins affect aligner grip and directionality.

The force decrease behavior observed in this study mirrors clinical experience with aligners exhibiting time-dependent relaxation. The exponential decrease in DPA_G_ closely resembles the stress-relaxation curve described in in-vivo evaluations [17], where forces typically drop significantly during the first 8–12 h. The observed decrease trajectory in DPA_G_ may reflect material- and trimline-dependent relaxation behaviour; however, targeted analyses would be required to isolate these effects mechanistically [12]. Interpretation of trimline- and material-related mechanisms in this study remains descriptive rather than causal, as pressure distribution, aligner-tooth contact behaviour and viscoelastic response were not separately quantified.

Interestingly, although DPA_G_ presented with increased material thickness in the palatal and incisal regions, it still generated lower forces compared to TFA_S_. This observation indicates that trimline geometry could influence force expression, potentially more than bulk thickness in this setting, although the relative contribution of geometry versus material behaviour warrants further investigation [21]. It also suggests that the effectiveness of DPA may not simply be a function of stiffness, but rather of controlled geometry and trimline compliance.

An important differentiation of TFA lies in the material structure. While Duran is a widely used single-layer PETG, newer multilayer foils such as CA Pro (Scheu-Dental GmbH, Germany), Erkodur-al (Erkodent Erich Kopp GmbH, Germany), and Zendura FLX (Zendura Dental, Bay Materials LLC, USA) aim to improve biomechanical behavior through enhanced force control and material resilience.

Lombardo et al. [22] and Cho et al. [23] found that multilayer materials exhibit more gradual stress relaxation and generate lower, more stable forces during tipping movements compared to monolayer PETG. Neoh et al. [24] further demonstrated that these multilayer foils offer improved mechanical retention and resistance to hydrothermal aging, while Elshazly et al. [25] confirmed their superior performance under thermomechanical stress.

These findings align with our observation that TFA, particularly those made from single-layer PETG, produced higher initial forces under identical activation conditions in this model. Multilayer materials mitigate this by distributing forces more evenly and degrading more predictably, though they still lack the design flexibility of DPA.

Overall, while multilayer TFA present a biomechanical improvement over traditional PETG, they remain constrained by the limitations of the thermoforming process. In contrast, DPAs allow precise control over force systems via custom geometry and trimlines - offering clear advantages in both force modulation and predictability.

The reduced transverse (Fy) and vertical (Fz) components in the DPA groups reflect more controlled resultant vectors in this in vitro setting; whether this corresponds to a lower risk of unintended root movement clinically remains to be validated in vivo. Such characteristics may increase treatment predictability and improve clinical outcomes [14, 26].

These results highlight a key benefit of additive manufacturing in orthodontics: the potential to tailor not only the global geometry but also the local biomechanics of aligners. Trimlines, surface offset, and selective thickening can all be individually controlled in the DPA workflow to generate specific force systems tailored to patient needs. In contrast, TFA production is largely constrained by uniform thermoforming sheets and limited control over fine structural features [3].

While the results of this study clearly favor DPA systems - particularly those with garlanded trimlines - it is essential to view these findings within the broader framework of aligner biomechanics. No aligner is biologically inert, and force systems are still susceptible to biological variability, intraoral conditions, and wear patterns. Nevertheless, this study offers insights that direct printed aligners offer superior mechanical control and adaptability.

### Limitations

This model measures the resulting forces and their components at a sensor unit; contact area or local pressure distribution on the tooth surface was not quantified, which may influence the transfer of force systems into clinical tooth movements. Furthermore, the simulation of viscoelastic and dynamic properties of the human periodontal ligament (PDL) was absent. Consequently, the absolute force values measured may differ from those occurring in vivo. Furthermore, intraoral factors such as saliva enzyme activity, occlusal loading, and temperature cycling were only partially simulated. The aligners were also not worn over extended durations, and patient-specific variation in bone density and morphology was not accounted for. Additionally, only early relaxation over 60 min was assessed; the findings should not be extrapolated to force behavior over typical clinical wear intervals (e.g., 24–48 h). Future studies should include long-term aging, multiple tooth movements, and clinical outcome comparisons.

## Conclusion

Direct-printed aligners with selective trimlines produced lower initial sagittal forces and smaller vertical components in this in-vitro model. Garlanded trimlines showed the lowest peak loads and most pronounced early force decrease. Further studies are needed to evaluate clinical relevance and long-term behavior.

## Data Availability

All data generated or analysed during this study are included in this published article.
